# A novel approach for assessing watershed susceptibility using weighted overlay and analytical hierarchy process (AHP) methodology: a case study in Eagle Creek Watershed, USA

**DOI:** 10.1007/s11356-019-06355-9

**Published:** 2019-09-06

**Authors:** Fadhil K. Jabbar, Katherine Grote, Robert E. Tucker

**Affiliations:** 1 Department of Geosciences and Geological and Petroleum Engineering, Missouri University of Science and Technology, McNutt Hall, 1400 N. Bishop Ave, Rolla, MO 65401 USA; 2 College of Science, University of Misan, Amarah, Iraq

**Keywords:** Susceptibility, Watershed health assessment, Analytic hierarchy process, Eagle Creek Watershed, Land uses, Weighted overlay analysis

## Abstract

Watershed vulnerability and the characterization of potential risk are important inputs for decision support tools in assessing watershed health. Most previous studies have focused on the assessment of the environmental risk using physicochemical properties of surface water and mathematical models to predict the health of a watershed. Here, we present a new methodology for evaluating watershed vulnerability using the analytic hierarchy process (AHP) and weighted overlay analysis. The new methodology provides an inexpensive approach for assessing areas that need more investigation based on known factors such hydrogeological, geological, and climate parameters without the need for site-specific physicochemical data. The proposed method was implemented using six main factors that influence water quality: land use, soil type, precipitation, slope, depth to groundwater, and bedrock type. Vulnerability was predicted for ten sub-watersheds within the Eagle Creek Watershed in Indiana using publicly available data input into geographic information system. Combination of watershed susceptibility assessment and GIS spatial analysis tools was used to produce the maps that show the susceptible zones within a watershed. A comparison of the resulting vulnerability estimates showed the expected significant positive correlations with measurements of nitrate, phosphate, temperature, and electrical conductivity. Likewise, the vulnerability estimates negatively correlated with dissolved oxygen and *E. coli*. Furthermore, the validation of the proposed approach revealed that the areas predicted to have high vulnerability did have lower water quality indices; the results showed a high negative correlation (*r*^2^ *=* 0.77, *p* < 0.05) between water quality index (WQI) and vulnerability which strongly suggests this method can be used successfully to assess a watershed’s susceptibility.

## Introduction

Water quality degradation from multiple sources of contamination has become a critical global issue (USEPA 2016; FAO 2017). Many water bodies across the USA are classified as impaired. Studies show that about 85% of streams and rivers and 80% of lakes and reservoirs are affected by nonpoint source (NPS) pollution (USEPA 2016), which can be attributed to sources such as agriculture and urbanization (Rowny and Stewart 2012; Liu et al. 2014; Ji et al. 2017). Agriculture can cause water quality degradation due to excess nutrients through fertilizers and manure (Kourgialas et al. 2017; Jabbar and Grote 2019) and runoff from pesticides and herbicides (Cruzeiro et al. 2015), as well as increasing turbidity level due to sedimentation and soil erosion (Zhang and Huang 2014). Numerous studies recorded the negative impacts of some agricultural practices on water quality (Dupas et al. 2015; Fournier et al. 2017). Likewise, urbanization affects the water quality through sediment, oils, and solid wastes washed from hard surfaces, bacteria, and input of nutrients from failing septic systems and wastewater (USEPA 2008; Walters et al. 2011; Zhao et al. 2015; Strangway et al. 2017).

Assessment of watershed susceptibility to contamination is an important step for decision making for sustainable environmental protection. As well as anthropogenic inputs, some features of the landscape or geologic conditions may make the watersheds more vulnerable to degradation. The vulnerability can be described as the level to which a system or system components are presumed to be impaired due to exposure to a potential risk or stress. Quantifying the vulnerability of watersheds to NPS pollution is important for recognizing which watersheds are most at risk of impairment and determining where changes in land use/land cover (LULC) might improve water quality conditions (USEPA 2008). Changes in land use, along with soil attributes, combined with topography, climate, hydrology, and other landscape variables are the most important factors contributing to a watershed’s quality (Bansal et al. 2014; Neupane and Kumar 2015; Fan and Shibata 2015; Serpa et al. 2017), so the watershed vulnerability assessment should be adaptable to potential changes. Hydrologists and environmental scientists are becoming increasingly aware and focused on the importance of identifying and quantifying risks to evaluate watershed’s health by using a convenient statistical technique and risk indicators. Therefore, the use of an appropriate model for watershed assessment that includes the variables listed above and can be modified as these variables change could be essential for evaluating continuous spatial and temporal distribution variations in watershed information. Some of these models are discussed in the following paragraph.

A number of methods have been developed to assess a watershed’s susceptibility to contamination using integrated watershed models and criteria evaluation methods (Sahoo et al. 2016; Ahn and Kim 2017; Kanakoudis et al. 2017). For example, the method for vulnerability mapping conducted by Tran et al. (2012) used the self-/peer-appraisal method and 50 variables collected by the US Environmental Protection Agency’s (EPA) Regional Vulnerability Program for 141 watersheds to map watershed vulnerability in the Northeast of the USA. In another study, geostatistical applications were used to assess the vulnerability of watersheds to chloride contamination in urban streams for seven sites within four watersheds in the Greater Toronto area (Betts et al. 2014). Simha et al. (2017) applied vulnerability assessment as a quantitative technique in the island of Lesvos in Greece, where a set of 25 indicators was used to identify the influence of strategic management on the vulnerability indices. Moreover, the US EPA has developed different approaches and tools to assess watershed susceptibility to surface water pollution. One of the best known of these tools is the WRASTIC method, which is based on seven parameters that affect the potential for pollution including presence of wastewater (*W*), recreational activities (*R*), agricultural activities (*A*), size of the watershed (*S*), transportation avenues (*T*), industrial activities (*I*), and the amount of vegetative ground cover (*C*) (USEPA 2000).

Modern geographical information system (GIS) tools are a powerful method for gathering, managing, and manipulating spatial analysis data. In addition, GIS can provide a more consistent environment for displaying the input datasets and the results have achieved by a model, which is more useful in a decision-making process. The external models which linked to GIS data provide a manageable way for combining and evaluating parameters such as slope, land use/land cover, and soil types (Wondrade et al. 2013; Yu et al. 2016).

One method of evaluating natural systems such as watersheds is to use multiple-criteria decision-making (MCDM) techniques. One of the most widely used MCDM techniques is the analytic hierarchy process (AHP) (Saaty 1980). This approach has many steps, including assigning the hierarchical structure, specifying and ranking the relative weights for both the criteria and sub-criteria, determining the weights of each substitute, and measuring the final score (Saaty 2008; Moeinaddini et al. 2010). Using GIS and AHP has been proven effective in analyzing natural hazards such as landslides and floods (Gamper et al. 2006; Fernández and Lutz 2010) and environmental studies (Ying et al. 2007; Rahman et al. 2014). The GIS-based and analytic hierarchy process has been applied by Koc-San et al. (2013) to choose a suitable site for an astronomical observatory. The same technique was used in Konya, Turkey, by Uyan (2013) to select the best site for solar farms. Likewise, Anane et al. (2012) applied this approach in the Nabeul-Hammamet region (Tunisia) to find suitable sites for irrigation with reclaimed water. Dong et al. (2013) used remote sensing, GIS, and AHP models to assess a habitat suitable for water birds in the West Songnen Plain in China.

In this research, we propose a new watershed susceptibility assessment method to evaluate watershed susceptibility to pollution using GIS and AHP methods. Six main factors have suggested in this study, which include land use/land cover, soil type, average annual precipitation, slope, depth to groundwater, and bedrock type. The general assumptions that were considered in this study of watershed vulnerability assessment are based on the response of watersheds to different contamination impacts and how the six factors work together to affect watershed health. This approach uses different databases to predict the NPS pollution in a watershed without field and lab work, which is a useful first approximation of vulnerability with minimal cost and time commitments.

## Study area

**Fig. 1:**
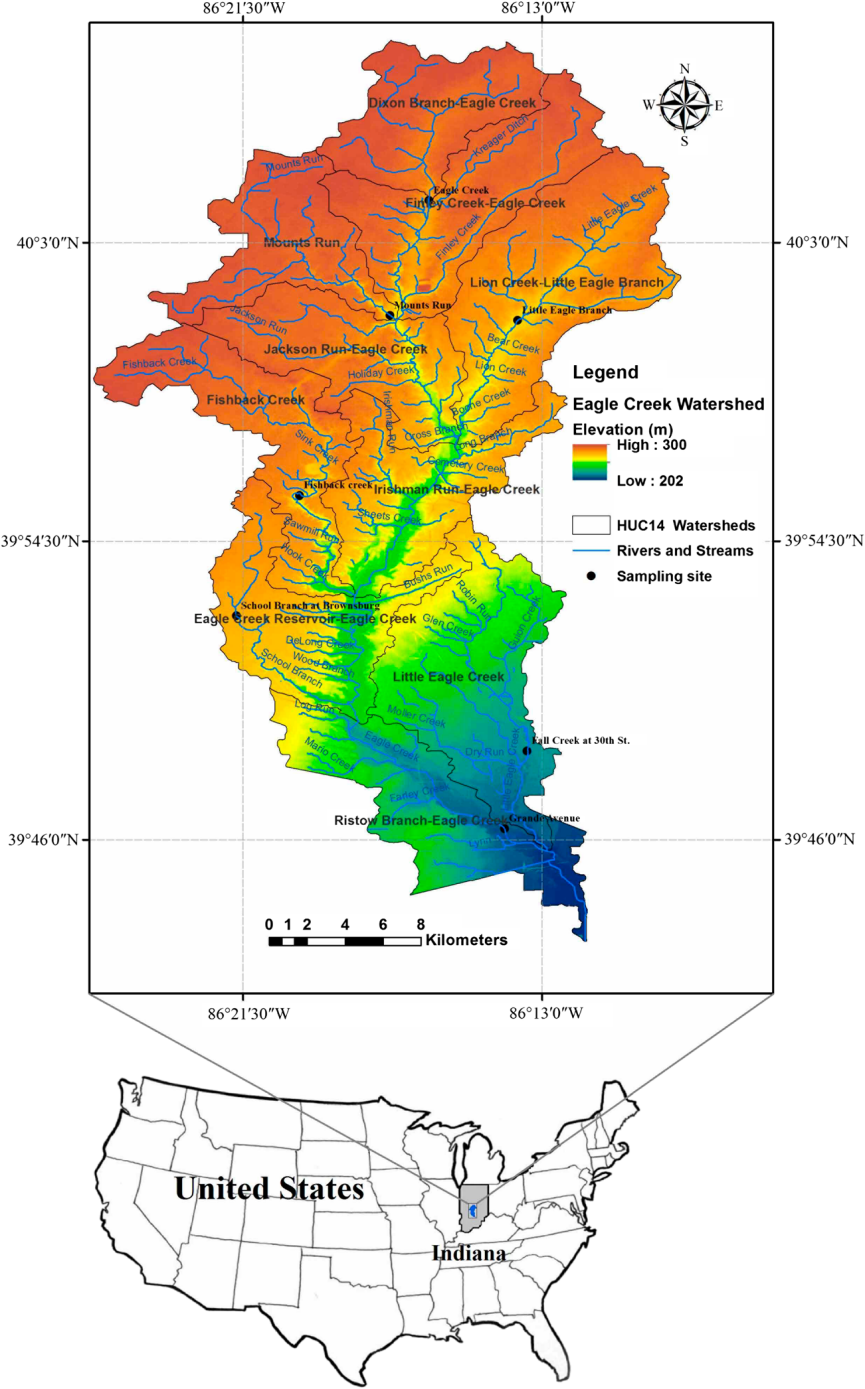
The location of the Eagle Creek Watershed

The Eagle Creek Watershed (ECW) is located in Central Indiana. The watershed is in the northern portion of the Upper White River Watershed that lies within the Mississippi River Basin (Fig. 1) and is a hydrologic unit code 14 (HUC14) level watershed. The drainage area is about 459 km^2^, and there are 10 sub-watersheds within the ECW varying in size from 26.9 to 70.7 km^2^ (Table 1). The ECW includes three main branches: School branch, Fishback Creek, and Eagle Creek branch, which flow into the Eagle Creek Reservoir. The Eagle Creek Reservoir is the main source of drinking water for Indianapolis. These branches are fed by eight main tributaries: Dixon Branch, Finley Creek, Kreager Ditch, Mounts Run, Jackson Run, Woodruff Branch, Little Eagle Branch, and Long Branch. The average flow distribution for the three major branches is around 2.85 m^3^/s for Eagle Creek and contributing about 80% of the water to the reservoir, while the average flow distributions for both Fishback Creek and School Branch are 1.1 m^3^/s and 0.5 m^3^/s, respectively, which contributes about 20% of water to the reservoir (Tedesco et al. 2005).

**Table 1:** Sub-watersheds and their drainage area in the Eagle Creek Watershed

Sub-watershed name	River or stream	Station name	Drainage area (km^2^)
Dixon Branch-Eagle Creek	Eagle Creek	Eagle Creek	42.5
Mounts Run	Mounts Run	Mounts Run	41.2
Finley Creek-Eagle Creek	Finley Creek	Finley Creek	26.9
Lion Creek-Little Eagle Branch	Little Eagle Branch	Little Eagle Branch	40.6
Jackson Run-Eagle Creek	Jackson Run		48.5
Fishback Creek	Fishback Creek	Fishback Creek	54.0
Irishman Run-Eagle Creek	Irishman Run		48.5
Eagle Creek Reservoir-Eagle Creek	School Branch	School Branch at Brownsburg	51.0
Little Eagle Creek	Little Eagle Creek	Fall Creek at 30th St.	70.7
Ristow Branch-Eagle Creek	Eagle Creek	Grande Avenue	35.1

The primary land use in the ECW is agriculture with approximately 56%, and 38% of the watersheds covered with urban land use, mostly in the southeast part of the watershed. The substantial majority of the remaining is either forested land or is grassland (USGS, National Hydrography Dataset). Precipitation is characterized by long-duration and moderate-intensity storms during the cooler months, and short-duration, high-intensity storms in the late spring and summer months. The average annual precipitation for the Eagle Creek Watershed is 1050 mm (Tedesco et al. 2005). According to the Midwestern Regional Climate Center (MRCC 2016), the lowest rainfall occurs in February, with an average of 59.7 mm and the highest rainfall occurs in May with an average of 115.5 mm. The watershed topography is relatively flat, with slopes less than 3%, especially in the agricultural areas. Steeper slopes are found adjacent to rivers and streams. Soils in the upper portion of the watershed consist of thin loess over loamy glacial till. These soils are classified as deep and poorly drained, but in the northwest part of the watershed, soils are poorly drained to well drained, while downstream areas are dominated by soils that are generally classified as deep and well drained to slightly poorly drained; soils were formed in a thin layer of silt and resulting from underlying glacial till (Hall 1999). The bedrock units of the Eagle Creek Watershed are generally characterized by brown, fine-grained dolomite and dolomitic limestone in the far northeastern part of the watershed, and sandy dolomitic limestone to brown sandy dolomite, but the southwest part is characterized by the gray, shaley fossiliferous limestone. The southern part of the watershed is dominated by brownish-black shale, greenish-gray shale, in addition to small amounts of dolomite and dolomitic quartz sandstone (Shaver et al. 1986; Gray et al. 1987).

## Materials and methods

### GIS data processing

**Fig. 2:**
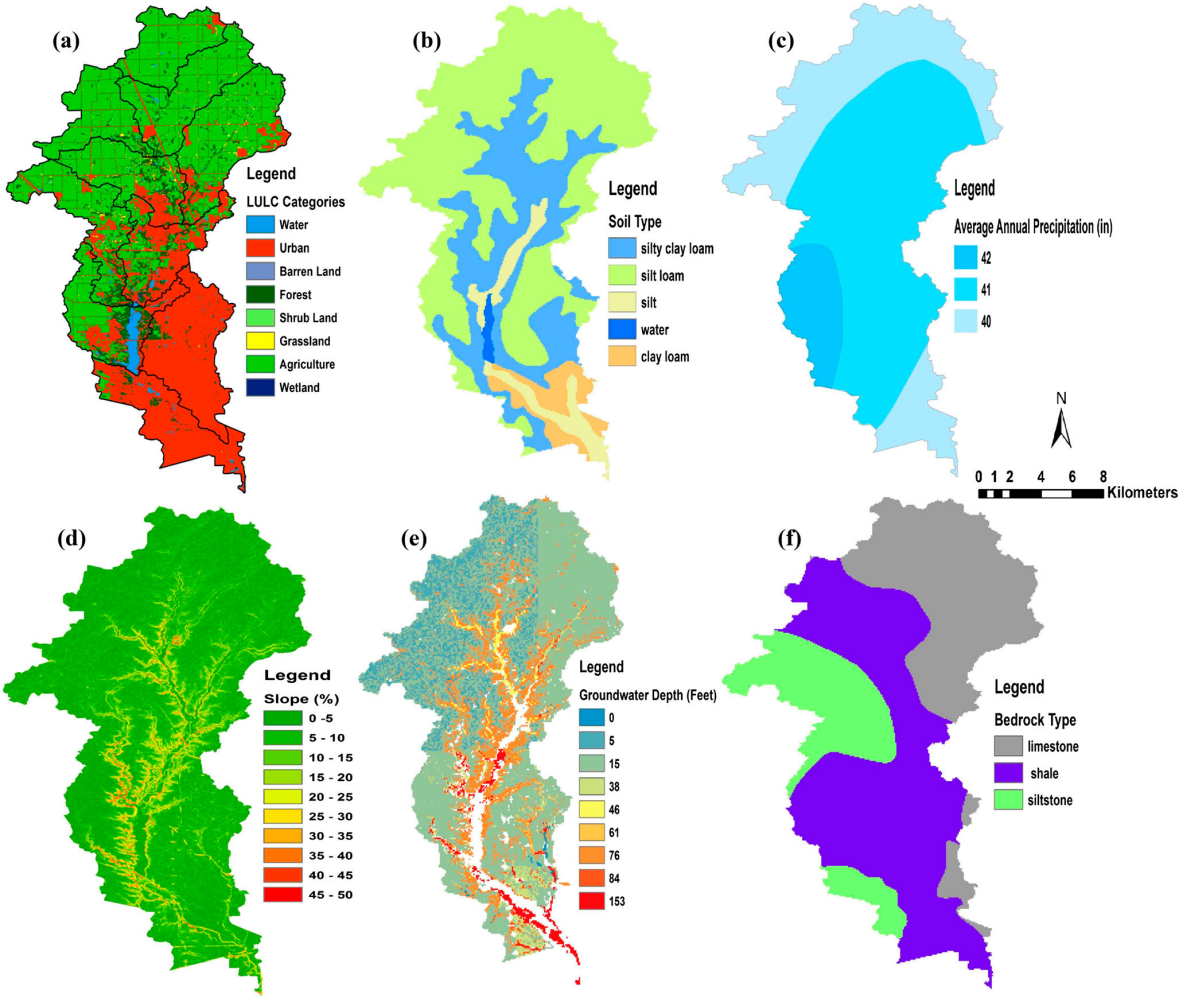
Thematic maps of the layers proposed for watershed susceptibility assessment method

Remote sensing data were used to create thematic maps for the proposed study area (Fig. 2). The general topographic surveying and mapping of the landscape features within the ECW were derived from a digital elevation model (DEM) has resolution (30 m) to investigate the important watershed characteristics, such as elevation variations and the slope of the land surface. The National Hydrography Dataset (NHD) and Watershed Boundary Dataset (WBD), which are managed by the USGS, were applied to calculate some watershed characteristics such as soil type, depth to groundwater, bedrock type, hydrologic units, catchment areas, and related features, including rivers and streams (USGS 2016). The National Land Cover Database 2011 (Homer 2015), which includes 15 LULC categories, was used for this study. Some of the LULC categories were combined to be more meaningful in this study. All categories labeled “developed” were aggregated into one class “urban”, and all categories labeled “forest” were aggregated into one class. Similarly, “wetland” categories were aggregated. GIS-based methods were applied to analyze the datasets and to determine the average values of parameters for each sub-watershed. The Parameter-elevation Regressions on Independent Slopes Model (PRISM) has been adopted to derive the average annual precipitation raster for the climatological data period 1961–1990 (Daly 1996).

### Water quality data

**Fig. 3:**
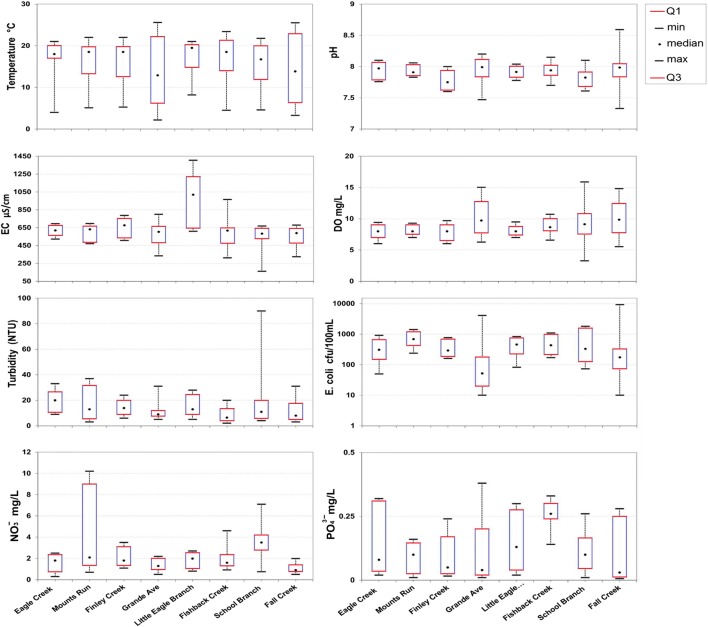
Boxplots showing the range of variations from minimum to maximum and the typical value (median) of water quality parameters

A statistical description of the water quality parameters which were measured by the Indiana Department of Environmental Management, Indiana Water Quality Atlas, (https://www.in.gov/idem/nps/pages/iwqa/index.html) is shown in Fig. 3. This figure shows that significant differences in water quality measurements occurred between sub-watersheds. Samples were collected from eight river stations which were treated as independent watersheds. Temperature and pH showed relatively little variation and are the most constant parameters within the study area. Dissolved oxygen (DO) showed a relatively slight variation for many sub-watersheds but was significantly higher in the Eagle Creek River at the Grande Avenue, School Branch, and Fall Creek stations. Electrical conductivity (EC) showed more significant variation between watersheds where the minimum value was observed between 160 and 640 μs/cm and the maximum value was between 523 and 1405 μs/cm. Results of turbidity reveal relatively little differences between all sub-watersheds, except the highest turbidity value was observed in the School Branch watershed (about 90 NTU). The measurements of *Escherichia coli* (*E. coli*), phosphate, and nitrate showed significant differences between sub-watersheds, where *E. coli* was somewhat higher in the southern part of the study area. Phosphate and nitrate concentrations are comparatively higher in northern sub-watersheds, where agricultural land is the most dominant land use.

## Methodology of watershed susceptibility assessment

### Analytical hierarchy process evaluation model

The AHP is an effective multicriteria decision-making tool that can be used to set a systematic approach for evaluating and integrating the impacts of different factors, which include some levels for qualitative and quantitative information (Saaty 1990). The AHP method can reduce problems between factors such as interrelationship and overlapping. The relative weight for each factor considered in this study was estimated using the methods of AHP and pairwise comparison matrix. The comparative scale (Saaty 1980) is a common methodology typically performed to analyze the comparison between various factors. The relative importance is measured between two factors based on a scale from 1 to 9, where 1 indicates the two factors are equally important while 9 reflects that one factor is much more important than another (Table 2). The consistency ratio (CR) was computed to check the differences between the pairwise comparisons and the reliability of the measured weights. The consistency ratio should be < 0.1 to be accepted; otherwise, it is important to check subjective judgments and recalculate the weights (Saaty and Vargas 2001).

**Table 2:** Judgments scale and definitions for the pairwise comparison

Qualitative definition	Explanation	Intensity of importance
Equal importance	Two activities contribute equally to the objective	1
Weak		2
Moderate importance	Experience and judgments slightly favor one activity over another	3
Moderate plus		4
Strong importance	Experience and judgment strongly favor one activity over another	5
Strong plus		6
Very strong or demonstrated importance	An activity is favored very strongly over another and dominance is demonstrated in practice	7
Very, very strong		8
Extreme importance	The evidence favoring one activity over another is of the highest possible order of affirmation	9

In current research, the decision-making problem structure was created and includes numbers that are symbolized by *m* and *n*. The values for both *a*_*ij*_ (*i =* 1, 2, 3…, *m*) and (*j =* 1, 2, 3..., *n*) are applied to find the performance values of matrix of *i*th and *j*th*.* The values of comparison criteria are utilized to fill out the upper diagonal of the matrix, while the lower triangular of the matrix is filled with the reciprocal values of the upper diagonal. In pairwise comparison matrix *A*, the element *a*_*ij*_ of the matrix is identified as the relative importance of the alternatives *i*th and *j*th with consideration to criterion *A* as shown below where *a*_*ji*_ is the reciprocal values of *a*_*ij*_.

The comparison matrix for any problem can be represented by the following decision matrix:

1 $$ A=\left(\begin{array}{cccc}1& {a}_{12}& \cdots & {a}_{1n}\\ {}1/{a}_{12}& 1& {a}_{23}& {a}_{2n}\\ {}\cdots & 1/{a}_{23}& \cdots & \cdots \\ {}1/{a}_{1n}& 1/{a}_{2n}& \cdots & 1\end{array}\right) $$

where *a*_*j*_; *I*, *j =* 1, 2, ……, *n* is the element of row *i* and column *j* of the matrix and equal to the number of alternatives.

The eigenvector was calculated for each row by using Eq. (2):

2 $$ {Eg}_i=\sqrt[n]{a_{11}\times {a}_{12}\times {a}_{13}\times \cdots \times {a}_{1n}} $$

where, *Eg*_*i*_ = eigenvector for the row *i*; *n* = number of elements in row *i.*

The priority vector (*Pr*_*i*_) was identified by normalizing the eigenvalues to 1 using the equation below:

3 $$ {\Pr}_i={Eg}_i/\left(\sum \limits_{k=1}^n{Eg}_k\right) $$

The lambda max (*λ*_max_) can be calculated from the summation of the results of multiplication of the priority vector and the summation of the column of the inverse matrix as shown below:

4 $$ {\lambda}_{\mathrm{max}}=\sum \limits_{j=1}^n\left({W}_j\times \sum \limits_{i=1}^m{a}_{ij}\right) $$

where *a*_*ij*_ = the sum of criteria in each column; *Wi* = the value of assigned weights for each criterion that is compatible with the priority vector in the decision matrix, where the values *i =* 1, 2, *… m*, *and j =* 1, 2, … *n.*

The consistency ratio (CR) was calculated by using the following equation:

5 $$ \mathrm{CR}=\frac{\mathrm{CI}}{\mathrm{RI}} $$

where CI is the consistency index can be determined according to the equation:

6 $$ CI=\frac{\lambda_{\mathrm{max}}-n}{n-1} $$

where *n* is the size of the matrix.

RI represents the random index that refers to the consistency of the pairwise comparison matrix which is randomly generated. It is obtained as the average of the random consistency index, which was computed by Saaty (1980) using a sample of 500 matrixes randomly generated. In the current study, weight scores for factors are obtained based on the AHP model (Table 3). A similar approach was applied to obtain rating values for individual sub-criteria used for watershed susceptibility assessment.

**Table 3:** A pairwise comparison matrix developed for assessing the relative importance of criteria for watershed susceptibility assessment

Factor	LULC	ST	BRT	Slope	AAP	DTG	Weights
LULC	1	3	4	5	3	2	0.36
Soil type (ST)	0.33	1	5	3	2	2	0.22
Bedrock type (BRT)	0.25	0.2	1	0.33	0.33	0.5	0.05
Slope	0.2	0.33	3	1	0.33	1	0.10
Average annual precipitation (AAP)	0.33	0.5	3	3	1	3	0.18
Depth to groundwater (DTG)	0.5	0.5	2	1	0.33	1	0.09
CR value = 0.02							

To calculate the watershed susceptibility values of the study area, the weighted overlay analysis was applied based on the following equation:

7 $$ \mathrm{WS}=\sum \limits_{j=1}^n{W}_j\times {C}_{ij} $$

where WS is the watershed susceptibility for area *i, W*_*j*_ is the relative importance weight of criterion, *C*_*ij*_ is the grading value of area *i* under criterion *j*, and *n* is the total number of criteria.

**Fig. 4:**
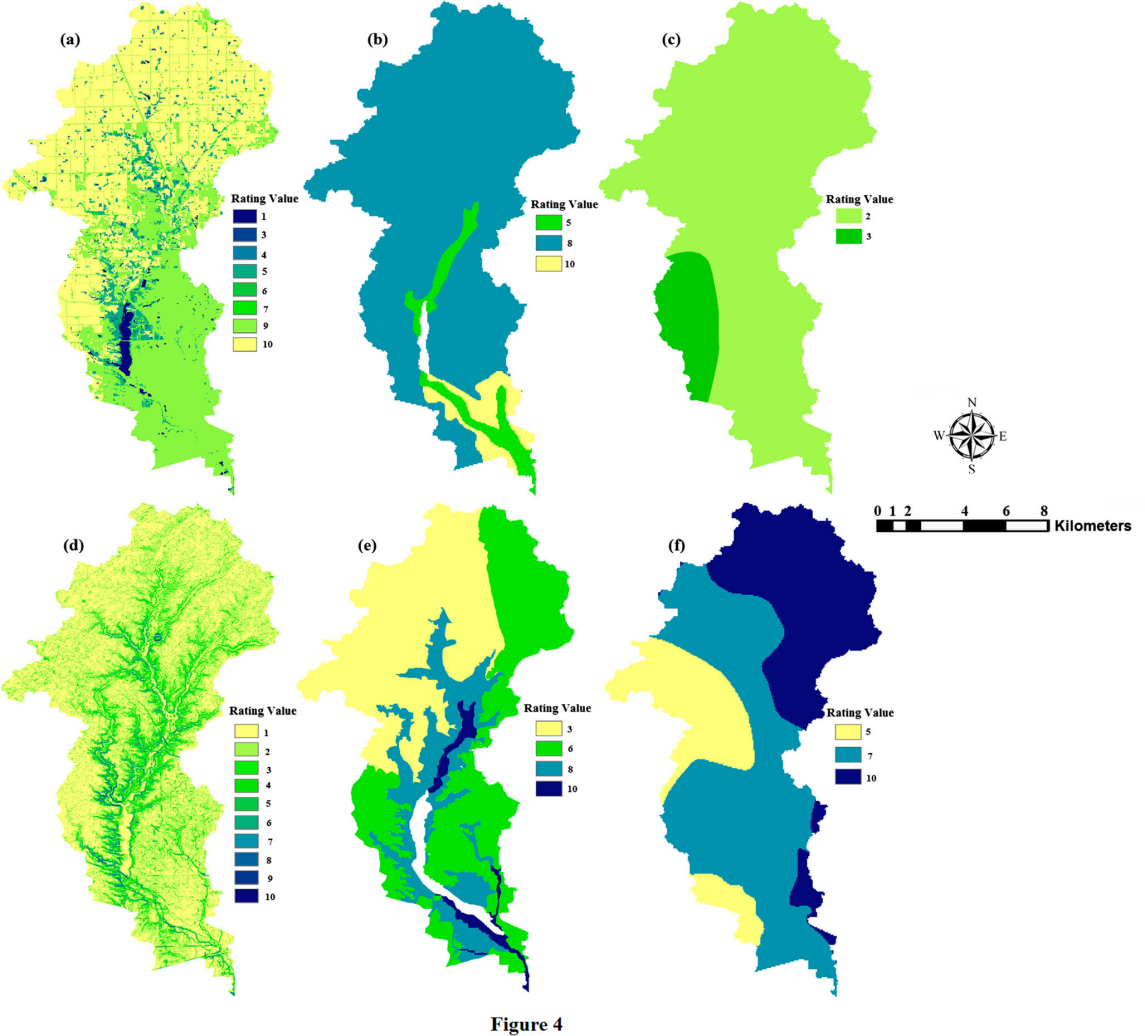
Thematic maps of the layers after rating for (a) land use/land cover, (b) average annual precipitation, (c) soil type, (d) slope%, (e) depth to groundwater, and (f) bedrock type

In this study, the assessment of a watershed’s susceptibility was the main objective for using the decision hierarchy. The process of hierarchy structure in the decision problem involves two steps. The first step has been classified into six factors: land use, soil type, precipitation, slope, depth to groundwater, and bedrock type. The second step includes 46 sub-categories used to evaluate the watershed’s health. For this study, according to the judgment of experts and literature reviews in this field (Eimers et al. 2000; Lopez et al. 2008; Xiaodan et al. 2010; Furniss et al. 2013; USEPA 2013; Shao et al. 2016; Siqueira et al. 2017), in addition to the data available and required for the study area, each factor was categorized into classes (sub-category). Then, each sub-category was specified for a suitability rating value. After creating these factors, the maps which are required for each layer were obtained as a shapefile (vector) or raster. Shapefile maps were then converted to raster maps to be more useful in reclassifying sub-categories based on the new rating, as illustrated in (Fig. 4). To prepare each category and sub-category, a number of steps were implemented using ArcGIS 10.5 software (i.e., overlay, convert, reclassify, and raster calculator). Output watershed susceptibility map is carried out by calculating the weighted overlay of the land uses/land cover, soil type, average annual precipitation, slope, depth to groundwater, and bedrock type.

### Factors used for watershed susceptibility assessment

To assess the watershed susceptibility to pollution, six main factors have been used in this study: land use, soil type, average annual precipitation, slope, depth to groundwater, and bedrock type. The determination of factors, the development of ratings for each, and the ranking of the weights were based on a synthesis of previous studies which were conducted to investigate possible factors and their impacts on the surface water quality (Eimers et al. 2000; Lopez et al. 2008; Xiaodan et al. 2010; Furniss et al. 2013; USEPA 2013; Shao et al. 2016; Siqueira et al. 2017) as well as evaluation of factors correlating with environmental degradation in similar Midwestern watersheds (Hoorman et al. 2008; Jabbar and Grote 2019). Virtually, all of these factors have been demonstrated to impact surface water quality and change essential chemical properties of the water within the watershed. The general assumptions were considered in the study of watershed vulnerability based on the response of a watershed systematically to different contamination impacts and how the six factors working together can affect the watershed’s health.

For each of the factors discussed below, the boundaries of each sub-watershed were determined using the Watershed Boundary Dataset, maintained by the USGS. These boundaries were used for further GIS-based analysis on different data sets. The origins of each data set and manipulations of these data sets to obtain the desired parameters are described below.

#### Land use/land cover

Watershed health is susceptible to LULC. Therefore, LULC has been regarded as one of the most important factors having an effect on water quality (Mouri et al. 2011; Yu et al. 2016; Ding et al. 2016). LULC can impact surface water quality as point source and nonpoint source pollution. Generally, agricultural land use is the main provenance of NPS pollution, particularly nitrogen (N) and phosphorus (P), on surface water quality (Hoorman et al. 2008; McCarthy and Johnson 2009). Urban lands are also reported to have considerable effects on surface water quality because of the significant load of contaminants from the point and nonpoint sources (Mallin et al. 2008). The contamination from nutrients, organic matter, and bacteria originates mainly from waste produced by municipal wastewater treatment plants and undefined anthropogenic sources (Glińska-Lewczuk et al. 2016). In this study, the LULC (as determined from the National Land Cover Database 2011 (Homer 2015)), has been divided into eight classes based on their impact on watershed health, where agriculture land uses that have a high impact were classified and rated by a value of (10), while “water” land use class was classified as the lowest rating (1) (Table 4).

**Table 4:** The relative weights and rating scores of the factors and sub-criteria used for watershed susceptibility assessment

Factor	Weighting	Sub-criteria	Rating
LULC	0.36	Agriculture	10
		Urban	9
		Grassland	7
		Wetland	6
		Forest	5
		Barren land	4
		Shrubland	3
		Water	1
Soil type	0.22	Clay loam	10
		Silty loam	8
		Silty clay loam	7
		Clay	6
		Silt	5
		Sandy loam	4
		Peat	3
		Sandy	2
Average annual precipitation (inch)	0.18	> 75	10
		71–75	9
		66–70	8
		61–65	7
		56–60	6
		51–55	5
		46–50	4
		41–45	3
		35–40	2
		< 35	1
Slope (degree)	0.10	> 60	10
		31–60	8
		16–30	6
		11–15	4
		4–10	2
		< 3	1
Depth to groundwater (feet)	0.09	< 5	10
		5–10	8
		11–15	6
		16–20	5
		21–25	4
		26–50	3
		51–100	2
		> 100	1
Bedrock type (depth (0–50 ft))	0.05	Limestone	10
		Dolomite	9
		Shale	7
		Claystone	5
		Sandstone	3
		Metamorphic/igneous	1

#### Precipitation

Many studies have assumed that there is a direct relationship between precipitation and increasing pollution levels in surface water. Rapid precipitation can correspond to degradation in water quality of streams and rivers through surface runoff of pollutants (Mallin et al. 2008; Whittemore 2012; Scott and Frost 2017). The high rating of precipitation with watershed susceptibility is associated with rainfall magnitude and intensity due to their impact on sediment and nutrient loading. Therefore, the precipitation (as obtained from the Midwestern Regional Climate Center (MRCC 2016)) was divided into ten classes, where the high rating (> 75 in) is represented by a value of (10), while the low precipitation had a value of (1) (Table 4).

#### Slope

Slopes that receive rapid precipitation play a significant role in affecting surface water quality (Chang et al. 2008; Qinqin et al. 2015; Meierdiercks et al. 2017). With a steep slope, this factor can increase the flow rate of a water body which can be causing soil erosion and sedimentation and carrying different kinds of pollutants like nutrients, pathogens, and pesticides to nearby rivers (Aksoy and Kavvas 2005; Bracken and Croke 2007). The eroded soil particles can be carried to rivers, which contribute to the level of total suspended solids and a decline in the water quality. Moreover, high slopes have a significant effect on the infiltration rate to groundwater, where the amount of infiltration decreases with the increase in the slope (Fox et al. 1997). Therefore, this study suggested six classes of slope based on their impact on the amount of rainfall that flows over the land surface as overland flow and reaches to surface water or contributes to groundwater by infiltration. Gentle slopes are represented by a value of (1), while steep slopes are classified as having a high value (10) (Table 4), because steep slopes can increase surface runoff that may cause soil erosion and carries different types of pollutants. The average slope for each sub-watershed was determined using a digital elevation model (DEM) has resolution (30 m) obtained from the National Hydrography Dataset (NHD) and slope-calculating algorithms in ArcGIS.

#### Depth to groundwater

Surface water and groundwater are connected through a wide range of catchment processes (Dahl et al. 2007; Lehr et al. 2015). Geological factors contribute to groundwater quality, mainly through the influence of chemical processes of water-rock interaction. Therefore, there is a significant impact of rock and soil components on the evolution of water quality by changing the chemical and physical properties of water (Varanka et al. 2014). During rainfall periods, much of the water that flows into nearby rivers and streams comes from shallow pathways through macropore flow in the soil zone, when infiltration to the aquifer is a substantial quantity. The water table will rise to the surface and seep from groundwater into the river, where surface water mixes with groundwater in the hyporheic zone (Lautz and Siegel 2006). The depth to groundwater was classified for eight classes where the shallow groundwater was classified as having a high rating (10), but the deep groundwater was identified as a low rating (1) (Table 4). The depth to groundwater for each watershed was determined by using maps generated by the National Hydrography Dataset (USGS) and calculating the average depth to groundwater for each sub-watershed using GIS tools.

#### Bedrock type

Water quality is typically greatly affected by different types of geologic materials, such as sedimentary, igneous, metamorphic rocks, and glacial deposits. Long-term geochemical interaction (rock–water) due to different chemical processes can occur between groundwater and aquifer materials (Oelkers and Schott 2009; Walter et al. 2017). When water flows through fractured rock aquifers (e.g., limestone or dolomite), the chemical properties of groundwater can be significantly changed because of the dissolution of some carbonate and evaporite minerals in the aquifer. Therefore, the quality of surface water can be affected by the exchange of water between rivers and shallow aquifers, especially in the alluvial aquifer. Water can seep from a shallow aquifer into the adjacent river and river water flows into the shallow aquifers alternately, depending on the oscillating of water table and river stage. In our study, rock types have been classified for six classes based on their resistance to weathering. The class of metamorphic/igneous rocks was given a low value (1), as this type of rock is normally very hard and resistant to weathering, while limestone was given a high rating (10) (Table 4). The bedrock type for each sub-watershed was determined using the National Hydrography Dataset (USGS) and averaging the values for each bedrock type based on the number of pixels associated with each bedrock type within each sub-watershed.

#### Soil type

Soil can be a source of soluble materials and suspended sediments (Kerr 1995). In general, sediment is the water pollutant which mostly affects surface water quality biologically, physically, and chemically (Rickson 2014). Bigger, heavier sediments like pebbles and sand settle first while smaller, lighter sediment particles like silt and clay can stay for a long time, increasing water turbidity. Furthermore, many types of soluble salts in the soil can affect water quality by increasing electrical conductivity (EC) (Chhabra 1996). A high clay content will increase EC due to the high cation-exchange capacity (CEC) of clay minerals. Soil types have been classified for eight soil classes based on their impact on water quality. The sandy type of soil was given a low value (1), while clay loam was classified and given a value of (10) (Table 4), since this soil type can affect water quality by increasing turbidity and salinity. The soil type for each sub-watershed was determined using the National Hydrography Dataset (USGS) and averaging the values for each soil type based on the number of pixels associated with each soil type within each sub-watershed.

## Results and discussion

The watershed susceptibility assessment method uses some features that have been weighted based on their contribution in surface water contamination and calculates a vulnerability index value for the area under consideration. The vulnerability to pollution is ranked as follows: for values of 70–100, watershed vulnerability is very high; values of 50–70 is high vulnerability; values of 30–50 is moderate vulnerability; values of 10–30 are low vulnerability; and values of 0–10 are very low vulnerability to contamination. To implement the proposed method, six main factors have been identified to evaluate ten sub-watersheds within the ECW. Assessment units ranked between 0 and 1 have low scores—indicating a very low impact on water quality. High scores were assigned as having a very high impact on water quality. Sub-categories were rated between 1 and 10 where 1 refers to very low impacts on water quality while high scores generally were rated as having a very high impact.

**Fig. 5:**
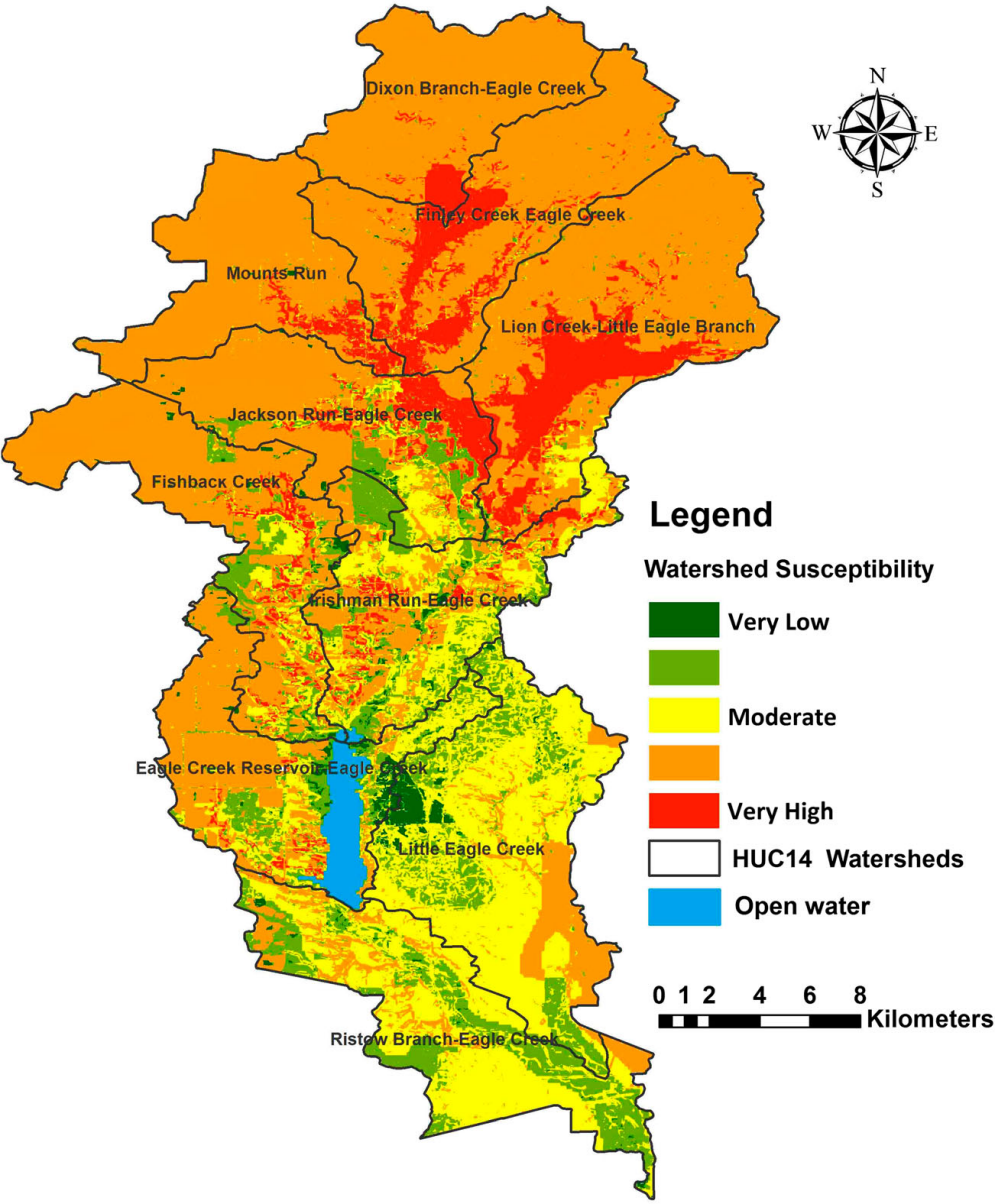
Watershed susceptibility distribution map of the Eagle Creek Watershed

The vulnerability evaluation of each watershed was used to create maps showing relative vulnerabilities of sub-watersheds. The map of watershed susceptibility in Fig. 5 shows a remarkable difference between the sub-watersheds in the vulnerability to pollution in the ECW. The upper part of the watershed, represented by Lion Creek and Finley Creek sub-watersheds, has been classified as likely to have very high vulnerability to potential contaminants. Similarly, the sub-watersheds Dixon Branch, Mounts Run, and Jackson Run are also identified as highly vulnerable to contamination based on the average value of vulnerability. Thus, around 37.6 km^2^ (8%) of the total area of the ECW was classified as having a very high vulnerability to contamination, and 284.5 km^2^ (57%) as a high vulnerability. The greatest area of contamination vulnerability is located in the north and middle of the study area where agricultural land comprises nearly 85% of total area within the northern sub-watershed. The low and very low range of vulnerability occupies an area around 73.8 km^2^ (14%) and 7.3 km^2^ (1%), respectively.

The results showed that very high vulnerability zones were located along the Little Eagle Creek, Finley Creek, Dixon Branch, and Mounts Run Creek. Agriculture is the main land use in this part of the study area, so the high vulnerability in this area is partially caused by agricultural runoff. In addition, the soil type could be another factor influencing water quality. Silty clay loam was the most common type of soil around the drainage channels in the northern part of the ECW. The steepest slopes in this part of the study area are also located near riverbanks. Therefore, the slope factor can increase both the surface runoff rate and soil erosion, increasing the delivery of sediments and pollutants to nearby streams (Tedesco et al. 2005). This process probably causes a deterioration of water quality by increasing electrical conductivity due to the solubility of the lime and soils that contain salts. Moreover, the type of bedrock (limestone), which is close to the surface in northern watersheds, can also lead to a declining water quality by increasing the electrical conductivity of groundwater due to the rock–water interaction in the aquifer (Walter et al. 2017). Eventually, this may later influence surface water quality through local exchange between streams and adjacent shallow aquifers (Lautz and Siegel 2006). The electrical conductivity of groundwater ranged between 500 and 1000 μs/cm in many parts of the ECW. It is evident that the high values of salinity which are observed in many study area streams are likely to be a significant indication of surface water-groundwater interaction.

The vulnerability of the watersheds in the southern part of the study area was classified between medium and weak, especially in the adjacent portions of sub-watersheds along School Branch, Eagle Creek at Grande Avenue, and Little Creek at the 30th Street. Bacterial contamination (*E. coli*) is the main source of degradation in water quality in the southern part of the watershed, where the urban development is the primary land use. The urban surface runoff can carry considerable quantities of contaminants, including major nutrients and bacteria to nearby streams (Tetzlaff et al. 2010; McGrane et al. 2014). The high levels of *E. coli* that were observed in the study area may explain the negative impact of urban lands on water quality.

## Validation and sensitivity analysis of a developed method

**Fig. 6:**
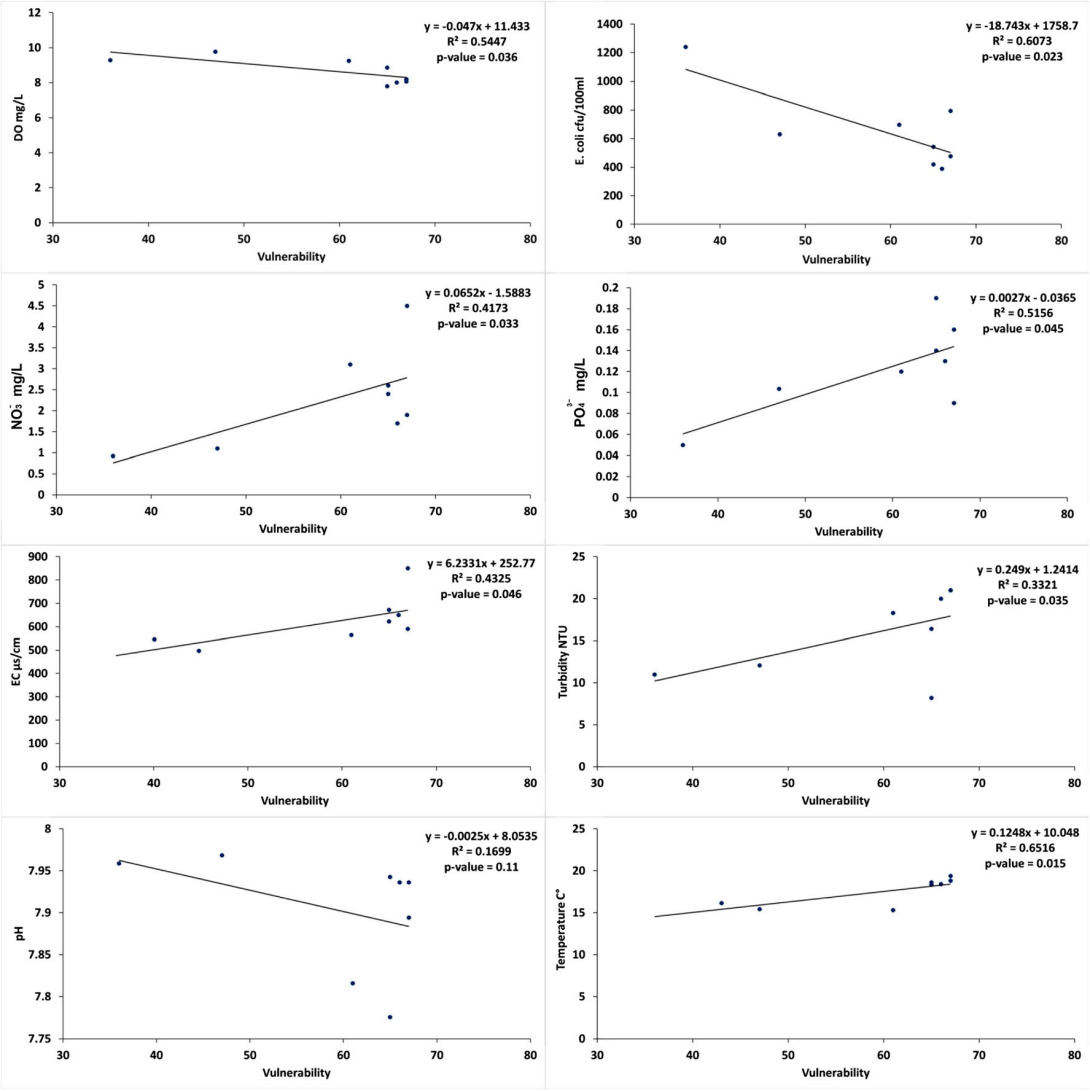
The relationship between watershed vulnerability and water quality parameters for ECW

The sensitivity of the new method of calculating vulnerability was evaluated by comparing the vulnerability rating to different water quality parameters. The correlation coefficients between water quality parameters and vulnerability results are shown in Fig. 6. These results show that the relationship between water quality and vulnerability was a significant positive correlation with phosphates (*r*^2^ *=* 0.5, *p =* 0.04), nitrates (*r*^2^ *=* 0.4, *p =* 0.03), and electrical conductivity (*r*^2^ *=* 0.4, *p =* 0.04). This indicates the vulnerability would increase with increasing concentrations of these parameters, which have been identified as the main parameters affecting water quality in the study area. The correlation coefficients for dissolved oxygen (*r*^2^ *=* 0.54, *p =* 0.036) and *E. coli* (*r*^2^ *=* 0.6, *p =* 0.02) have shown a significant negative relationship with vulnerability. This indicates the potential for water quality degradation as a result of high concentration of bacteria and low levels of dissolved oxygen in the southern part of the study area. Generally, in most watersheds of this study area, the *E. coli* levels were more than the acceptable limit, but the highest level of these bacteria was observed in the southern region which is dominated by urban development. However, the negative relationship between *E. coli* and vulnerability reflects the impact of land use type on water quality, where *E. coli* and DO seems to be highly associated with urban land use while *N* and *P* associated with agriculture land use.

To assess the water quality of streams and rivers in Eagle Creek Watershed, the water quality index (WQI) (Eq. 8) was applied based on the method which was developed by Cude (2001). The WQI is according to the sub-index measurements of water quality parameters that provide a summary of water quality on a rating scale from (0) very poor–(100) excellent.

8 $$ \mathrm{WQI}=\sum \limits_{i=1}^n{\mathrm{SI}}_i{W}_i $$

where WQI is Water Quality Index, SI is sub-index *i*, and *W*_*i*_ is the weight given to sub-index *i.*

**Fig. 7:**
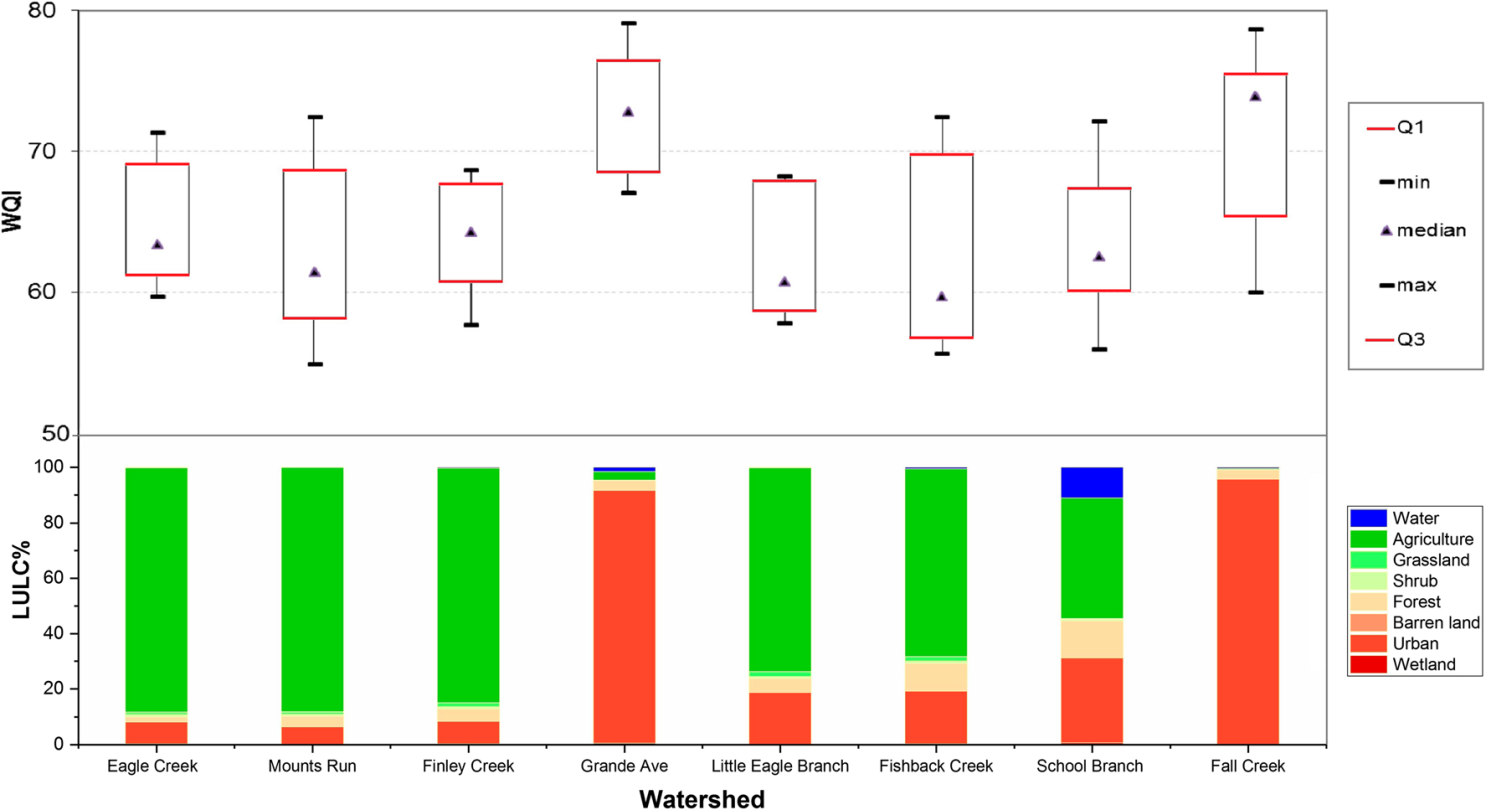
The relationship between land use/land cover (LULC) types and the WQI in the study area

**Fig. 8:**
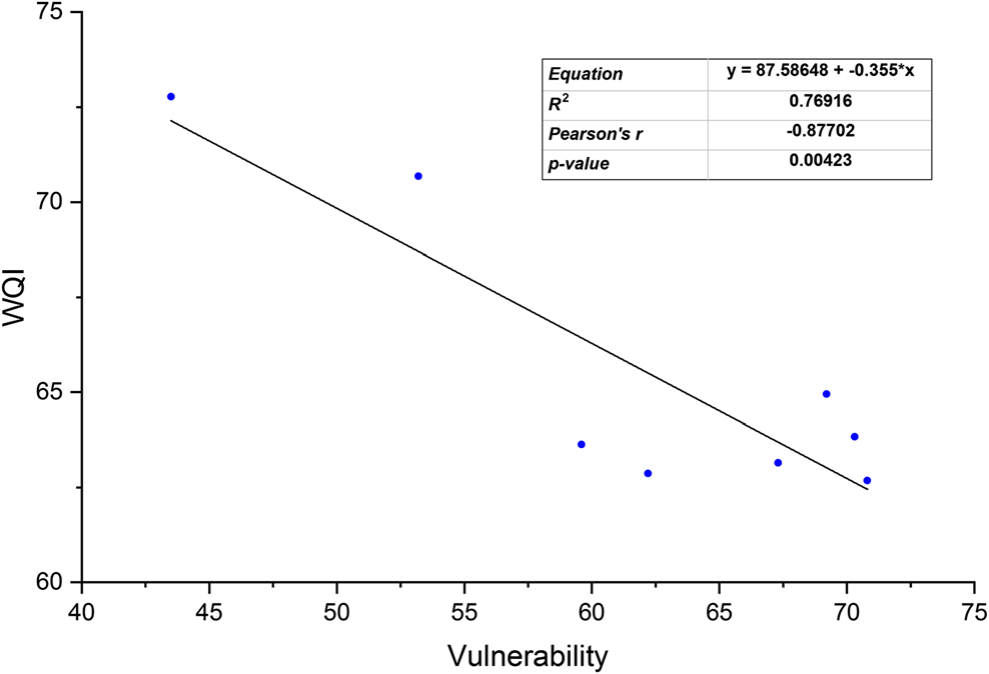
Comparison showing the relationship between watershed vulnerability and WQI

**Fig. 9:**
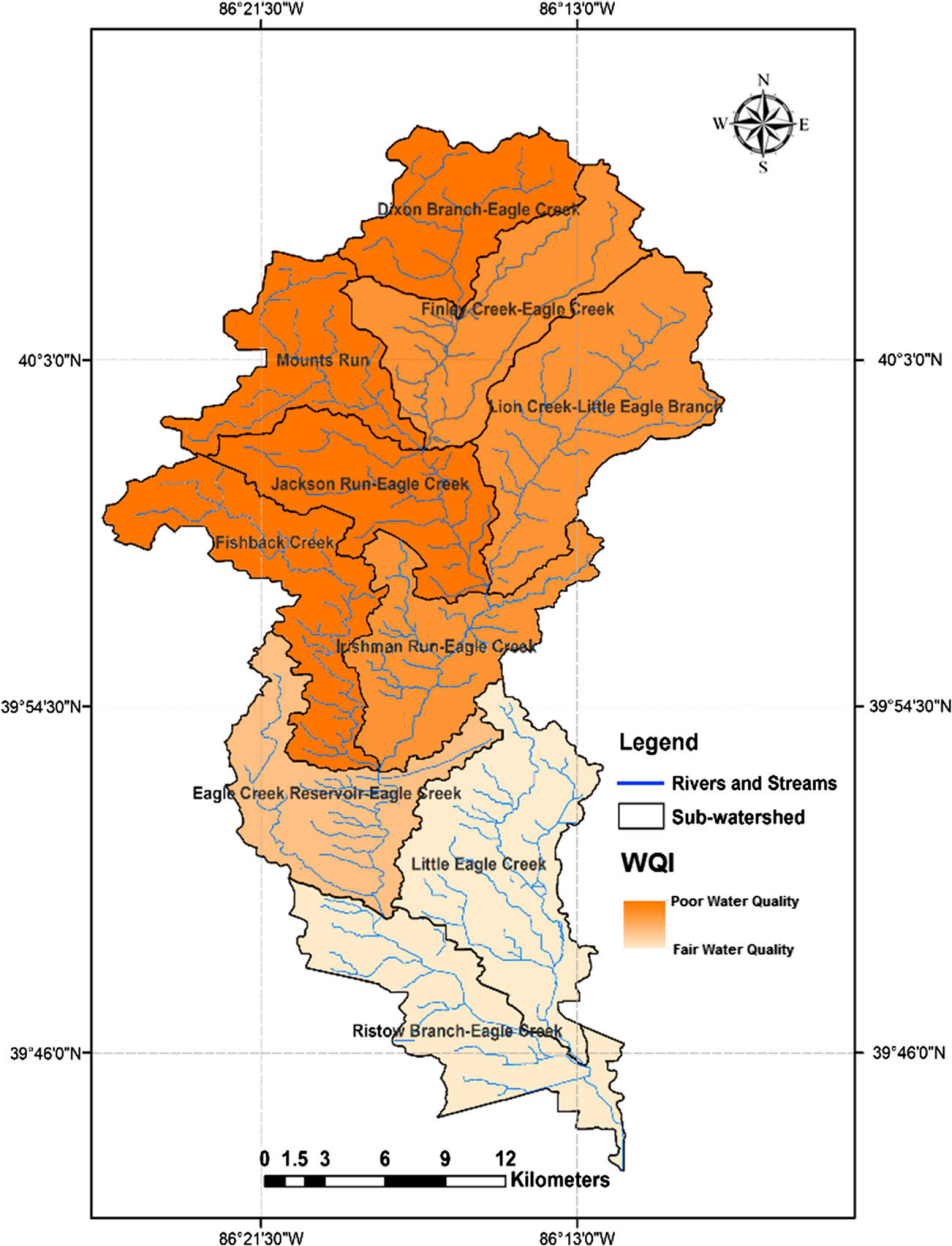
Spatial distribution of Water quality index (WQI) in the ECW

Based on the Water Quality Index results for all eight monitoring stations, it can be concluded that the Eagle Creek Watershed ranged between poor and fair in water quality. All water quality ratings within the northern sub-watershed were poor water quality. This indicator showed fair water quality in Fall Creek and Eagle Creek at Grande Avenue, all of which are located in the southern part of the watershed. In general, *E. coli*, nitrate, phosphate, and electrical conductivity are the most important parameters that influence surface water quality of these eight sub-watersheds. As can be seen from Fig. 7, as regards the comparison between the WQI and LULC, the surface water quality in the central and northern portion of the study area is classified as poor quality probably because the vast majority of land is agriculture. Conversely, the southern part of the study area shows fair water quality, where the land uses are dominated by urban land. The results of WQI which have been described above were adopted to emphasize the efficiency of the suggested method. As illustrated in Fig. 8, the correlation coefficients between the WQI and watershed vulnerability showed a significant high negative correlation (*r*^2^ *=* 0.77, *p <* 0.05). The results of WQI reflect the conditions of water quality in the study area which have been classified as very poor water quality (highly vulnerable to pollution) in the northern sub-watersheds, while it rated as moderate water quality (weak-moderate vulnerability) at the southern sub-watersheds as shown in Fig. 9. These results provide considerable evidence for adopting this method to assess a watershed’s susceptibility.

As a comparative study, Eimers et al. (2000) from the USGS developed a method to evaluate the unsaturated zone and watershed characteristics to predict potential contamination for both public groundwater and surface water supplies. This method was applied in North Carolina to evaluate around 11,000 public groundwater supply wells and around 245 public surface water locations. Some watershed characteristics were assigned based on their contribution to the potential that water (with or without pollutants) may reach a surface water supply through overland flow paths or shallow sub-surface flow paths. Factors identified for assessing unsaturated zone vulnerability are vertical hydraulic conductivity, slope, land cover, land use, average annual precipitation, and groundwater contribution. They suggested using statistical analysis of water quality measurements to refine and enhance factor weights and ratings, while in the current study, weight and rating scores were assigned by using the AHP model; additionally, statistical analysis was applied to validate the proposed method. In a recent study conducted by Arriagada et al. (2019) in the Andalién River watershed, located in Mediterranean Chile, they used a new method to evaluate the watershed vulnerability index (WVI) depending on three sub-indices includes environmental fragility, anthropogenic stressors, and natural disturbances. The results of WVI revealed the negative impacts of these stressors on watershed quality. The application of statistical analysis of water quality parameters was presented in the work of Arriagada et al. (2019) and in the current paper, the statistical analysis was applied along with WQI and the vulnerability levels to emphasize the efficiency of the suggested method.

## Conclusions

In this study, we identified the primary parameters affecting watershed vulnerability and suggested new weighting factors for each parameter using AHP analysis. The proposed method was implemented using suitable six main factors (land uses, soil type, precipitation, slope, depth to groundwater, and bedrock type) to evaluate the watershed susceptibility for 10 sub-watersheds within the Eagle Creek Watershed, Indiana. Combination of watershed vulnerability assessment and GIS spatial analysis tools was used to produce the maps that show the susceptible zones for watershed. Based on the results of this method, accounting for around 37.6 km^2^ (8%) of the total area of the watershed was classified as having a very high vulnerability to contamination, and 284.5 km^2^ (57%) as having a high vulnerability. The greatest portion of weakness is located in the middle and north of the study area where agricultural land takes up nearly 85% of the total area of northern sub-watershed, while the vulnerability for the watersheds in the southern part of the study area was classified between medium and weak. Regression relationships were used to test the effectiveness of this new method. The results demonstrated that the relationship between water quality and vulnerability was a significant positive correlation with phosphates (*r*^2^ *=* 0.5), nitrates (*r*^2^ *=* 0.4), and electrical conductivity (*r*^2^ *=* 0.43). The values of dissolved oxygen (*r*^2^ = 0.54) and *E. coli* (*r*^2^ = 0.6) have shown a significant negative relationship with vulnerability. The correlation between the measured water quality index and the predicted watershed vulnerability for the method showed a high negative correlation (*r*^2^ = 0.77) between WQI and vulnerability, indicating that the vulnerability predictions are fairly accurate. This method could be used in other watersheds to more accurately assess watershed susceptibility.
